# Real world analysis of treatment change and response in adults with attention-deficit/hyperactivity disorder (ADHD) alone and with concomitant psychiatric comorbidities: results from an electronic health record database study in the United States

**DOI:** 10.1186/s12888-024-05994-8

**Published:** 2024-09-16

**Authors:** Christian Liman, Jeffrey Schein, Ashley Wu, Xueyan Huang, Simran Thadani, Ann Childress, Scott H. Kollins, Sandipan Bhattacharjee

**Affiliations:** 1 Holmusk Technologies, Inc., Blk 71, Ayer Rajah Crescent, #06-07/08/09 and #07-08/09, Singapore, Singapore; 2 Otsuka Pharmaceutical Development & Commercialization, Inc., 508 Carnegie Center, Princeton, NJ 08540 USA; 3 Center for Psychiatry and Behavioral Medicine, 7351 Prairie Falcon Rd STE 160, Las Vegas, NV 89128 USA; 4 Holmusk Technologies Inc., 4th Floor, 54 Thompson St., New York, NY 10012 USA; 5 Department of Psychiatry & Behavioral Sciences, Duke University School of Medicine, Durham, NC 27705 USA

**Keywords:** Attention-deficit/hyperactivity disorder, Treatment change, Treatment response, Healthcare resource utilization, Electronic health records, Real-world data

## Abstract

**Background:**

The objectives of this study were to examine the association of psychiatric comorbidities and patient characteristics with treatment change and response as well as to assess the association between treatment change and healthcare resource utilization (HCRU) among adult patients with attention-deficit/hyperactivity disorder (ADHD) and psychiatric comorbidities.

**Methods:**

De-identified electronic health records from the NeuroBlu Database (2002–2021) were used to select patients ≥ 18 years with ADHD who were prescribed ADHD-specific medication. The index date was set as the first prescription of ADHD medication. The outcomes were treatment change (discontinuation, switch, add-on, or drop) and HCRU (inpatient, outpatient, composite) within 12 months of follow-up. Cox proportional-hazard model was used to assess the association between clinical and demographic patient characteristics and treatment change, while generalized linear model with negative binomial distribution and log link function was used to assess the association between key risk factors linked to treatment change and HCRU rates.

**Results:**

A total of 3,387 patients with ADHD were included (ADHD only: 1,261; ADHD + major depressive disorder (MDD): 755; ADHD + anxiety disorder: 467; ADHD + mood disorder: 164). Nearly half (44.8%) of the study cohort experienced a treatment change within the 12-month follow-up period. Treatment switch and add-on were more common in patients with ADHD and comorbid MDD and anxiety disorder (switch: 18.9%; add-on: 20.5%) compared to other cohorts (range for switch: 8.5–13.6%; range for add-on: 8.9–12.1%) Survival analysis demonstrated that the probability of treatment change within 12 months from treatment initiation in the study cohort was estimated to be 42.4%. Outpatient visit rates statistically significantly increased from baseline (mean [SD] 1.03 [1.84] visits/month) to 3 months post-index (mean [SD] 1.62 [1.91] visits/month; *p* < 0.001), followed by a gradual decline up to 12 months post-index. Being prescribed both a stimulant and a non-stimulant at index date was statistically significantly associated with increased risk of treatment change (adjusted hazard ratio: 1.64; 95% CI: 1.13, 2.38; *p* = 0.01).

**Conclusions:**

This real-world study found that treatment change was common among patients with ADHD and psychiatric comorbidities. These findings support the need for future studies to examine the unmet medical and treatment needs of this complex patient population.

**Supplementary Information:**

The online version contains supplementary material available at 10.1186/s12888-024-05994-8.

## Background

Attention-deficit hyperactivity disorder (ADHD) is a neurodevelopmental disorder that affects people of all ages and can interfere with daily functioning and quality of life [1]. ADHD symptoms can present as inattentiveness (e.g., difficulty paying attention, being easily distracted, forgetfulness, disorganization, difficulty completing tasks) or hyperactivity-impulsivity (e.g., fidgeting, difficulty sitting still, talking excessively, interrupting others, acting without thinking) [1]. Patients may experience any combination of these symptoms [1]. While estimates vary, the prevalence of adult ADHD in the United States (US) is estimated to be between 2% and 5% [2, 3], and recent research indicates that the incidence and prevalence of adult ADHD in the US are increasing [4]. 

Treatment for ADHD often involves a combination of pharmacologic (e.g., stimulant or non-stimulant medication) and non-pharmacologic interventions, such as cognitive behavioral therapy (CBT) [5–8]. Stimulants are the recommended first-line pharmacologic treatment option for adults and include amphetamine- and methylphenidate-based formulations [5, 9]. Clinical trials have shown that stimulants are effective at reducing behavioral symptoms for the majority of adult patients [10]. For patients who do not respond to stimulants or who experience tolerability issues, clinical guidelines recommend non-stimulant medication (e.g., clonidine, guanfacine, atomoxetine, or viloxazine) [5, 9]. Additionally, some medications may be used off label to manage ADHD symptoms, such as antidepressants or atypical antipsychotics [5]. In recent years, there has been a notable shortage in the US of front-line stimulant medication for ADHD treatment [11]. 

It has been shown that a substantial proportion of patients with ADHD may switch treatments to another ADHD medication or even discontinue treatment altogether [12, 13]. Common reasons for treatment discontinuation include a lack of symptom control, adverse events associated with drug therapy, dosing inconvenience, social stigma, and patient preferences [14, 15]. Some studies have shown that discontinuing medications increases the risk of exacerbation of ADHD symptoms and may be associated with a reduced quality of life in children and adolescents as well as high-risk behaviors [16, 17]. 

ADHD is primarily thought of as a disorder that affects children and adolescents and has typically been under-recognized and under-treated in the adult population [18, 19], although the incidence and prevalence have increased over the past decade [4]. This may be because adult ADHD often has a heterogeneous clinical presentation, which includes a wide spectrum of emotional dysregulation and functional impairment [18, 19]. Relative to healthy controls, adults with ADHD are more likely to have comorbid psychiatric disorders [19–21]. In a one-year post-market surveillance study of Japanese adults with ADHD treated with an osmotic-release oral system with methylphenidate, over half of patients (52%) had at least one diagnosed psychiatric comorbidity [22], and some estimates of coexisting psychiatric disorders among adult patients with ADHD in Europe reach as high as 80% [23, 24]. Common psychiatric comorbidities include major depressive disorder (MDD), anxiety disorder, eating disorders, and mood disorders [3]. Psychiatric comorbidities in adults with ADHD both increase the clinical complexity of illness and contribute to poor long-term outcomes [25]. Additionally, the presence of some psychiatric comorbidities may also adversely impact patient response to ADHD medications [26]. 

A recent US claims-based analysis found that patients with ADHD and comorbid anxiety and/or depression experienced statistically significantly higher odds of treatment change compared to patients with ADHD only [27]. The overall aim of this real-world study was to further understand how psychiatric comorbidities impact treatment change and response in adult patients with ADHD using electronic health record (EHR) data. The objectives of this study were to (1) examine the association of psychiatric comorbidities and patient characteristics with treatment change and response in adult patients with ADHD and (2) examine the association of treatment change with healthcare resource utilization (HCRU) among adult patients with ADHD.

## Methods

### Study design

This was a retrospective observational cohort study using de-identified EHR data.

### Data source

This study used de-identified EHRs from the NeuroBlu Database (Holmusk Technologies Inc., New York, NY, USA) Version 21R2. NeuroBlu is a longitudinal behavioral health real-world database comprising both structured and semi-structured patient-level clinical data aggregated from the MindLinc EHR [28]. At the time of the analysis, the database comprised over 560,000 patients and more than 14 million clinical visits at 25 hospitals/care systems in the US. The clinical sites included in this study were psychiatric specialty clinics. Thus, a combination of psychiatrists, nurses, or other mental health care specialists may have prescribed ADHD medications and conducted clinical assessments (e.g., Clinical Global Impression – Severity [CGI-S]) that were analysed in this study. The NeuroBlu Database has been standardized into a common data model (CDM) that conforms with the Observational Health Data Sciences and Informatics (OHDSI) data standards. For the MindLinc EHR, institutional review board approval for this study was not required because MindLinc data are de-identified and thus exempt from Health Insurance Portability and Accountability Act (HIPAA) requirements. The NeuroBlu Database platform received a waiver of authorization for analysis of de-identified healthcare data from the WCG Institutional Review Board (Ref: WCG-IRB 1-1470336-1).

### Study population

**Fig. 1:**
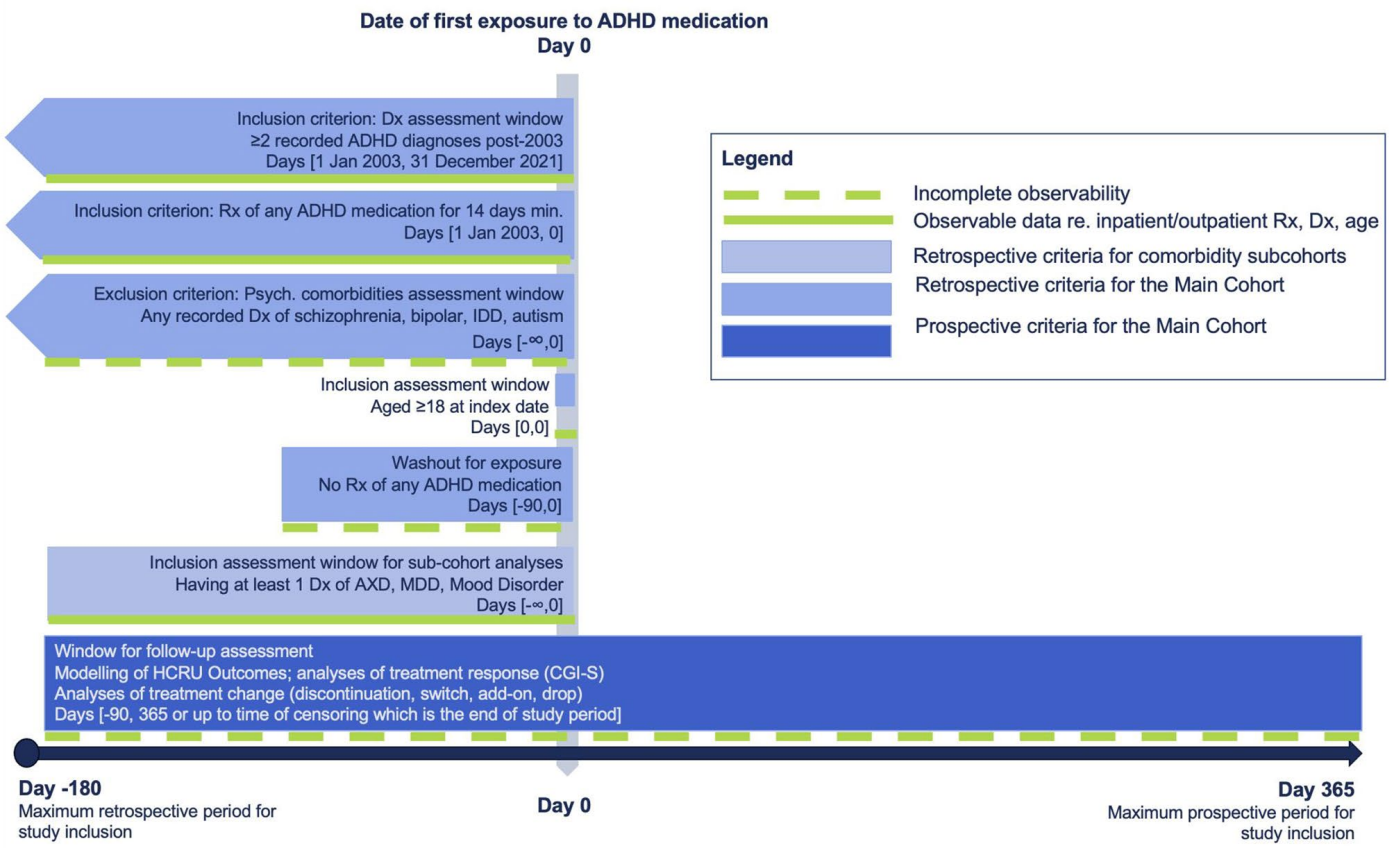
Study design **Abbreviations**: ADHD, attention-deficit/hyperactivity disorder; AXD, anxiety disorder; Dx, diagnosis; IDD, intellectual disabilities; MDD, major depressive disorder; Rx, prescription

Patients were included if they: 1) had ≥ 2 clinical encounters with a documented diagnosis of ADHD based on International Classification of Diseases, Ninth Revision, Clinical Modification (ICD-9-CM) of 314.00 or 314.01, or International Classification of Diseases, Tenth Revision, Clinical Modification (ICD-10-CM) of F90.0, F90.1, F90.2, F90.8, or F90.9; 2) were aged ≥ 18 years at first prescription of ADHD-related medication (index date; see Fig. 1); 3) were prescribed a pharmacologic medication for ADHD for ≥ 14 days (index event); 4) had a 90 day no ADHD-related treatment washout period (see Fig. 1) before their index prescription of ADHD-related medication; and 5) had ≥ 3 months of pre-index clinical activity, defined as a record of any type of visit. Patients were excluded if they had any lifetime diagnosis of schizophrenia, bipolar disorder, intellectual disabilities, or autism. Two subsets were derived from the study population (hereafter referred to as the Main Cohort) for specific analyses: (1) Subset A, which requires patients to have at least 12 months of post-index visit data, and (2) Subset B, which requires patients to have at least 6 months of post-index visit data.

### Study period and index date

This study period was set between July 1, 2002 to December 31, 2021. To allow variable assessment pre-index, index events were only allowed to occur between January 1, 2003 and December 31, 2021 (Fig. 1). The index date was defined as the date of first prescription of ADHD medication that satisfied the study eligibility criteria. The pre-index period was defined as 6 months (180 days) before the index date, while the follow-up period was defined as 12 months (365 days) after the index date.

### Variables and outcomes

Baseline variables included demographic (sex, age, race) and clinical characteristics (concomitant medications, ADHD symptoms, disease severity measurements [CGI-S], HCRU, and psychiatric comorbidities). CGI-S scores that were present ± 14 days from the index date were treated as baseline CGI-S scores. Baseline CGI-S scores were only reported on patients with CGI-S records within the aforementioned time window. For patients with multiple measurements, the median of all measurements at the measurement date closest to the time point of interest was used. ADHD symptoms that were present within ± 30 days from the index date were derived from MSE notes using previously published natural language processing (NLP) methods [29]. ADHD symptoms were only reported for patients with MSE records within the aforementioned time window. Index treatment characteristics were reported for the Main Cohort, including type of ADHD-related agent prescribed, treatment duration, and type of formulation. Pharmacological treatments for ADHD include short- and long-acting stimulants (amphetamine, dexmethylphenidate, dextroamphetamine, lisdexamfetamine, methamphetamine, methylphenidate, modafinil, and pemoline) and non-stimulants (clonidine, viloxazine, atomoxetine, and guanfacine).

Variables for outcome assessment included variables related to treatment changes from index ADHD medication (presence of any treatment changes, number of treatment changes, time to first treatment change, type of first treatment change), HCRU (time to first psychiatric hospitalization, inpatient psychiatric hospitalization frequency, emergency department [ED] visit frequency, outpatient visit frequency), and change in CGI-S. Treatment change was defined at the therapeutic agent level and includes the following four types (Supplemental Figs. 1–4): (1) Treatment discontinuation: defined as no ADHD-related agent for 120 consecutive days after last day of prescription of the index treatment regimen. Treatment discontinuation rate was determined by the percentage of patients who discontinued their index treatment regimen within the 12 months post-index follow-up period; (2) Treatment switch was defined as initiation of a new ADHD-related agent with no prescription refills from the index treatment regimen ± 60 days from the index treatment regimen end date. Treatment switch rate was determined by the percentage of patients who switched from their index treatment to a different therapeutic agent within the 12 months post-index period; (3) Treatment add-on was defined as initiation of a non-index ADHD-related agent that served as an adjunctive treatment with index treatment regimen; and (4) Treatment drop was defined as discontinuation of an ADHD-related agent for patients who had started with two or more ADHD medications at the index date (other medications prescribed from index, including another ADHD medication or non-ADHD medication, may have been continued). Frequency of visits were assessed by visit type (inpatient, outpatient, or ED) and estimated from the number of visits normalized by the length of the observation period (in months). In addition, a composite utilization rate, estimated using all inpatient and outpatient visits normalized by the length of observation period, was also reported to provide a comprehensive measure of overall HCRU. All utilization rates were calculated on a per-patient basis.

### Statistical analysis

#### Descriptive analysis of baseline characteristics

Continuous variables were summarized using means with standard deviations (SDs) for normally distributed data and medians with interquartile ranges (IQRs) for skewed data. Categorical variables were summarized using frequencies and percentages. Baseline comparisons between sub-cohorts for continuous data were made using Kruskal-Wallis *H* test, while comparisons for categorical data were made using Chi-squared test, or Fisher’s exact test if the counts in any of the sub-cohorts were deemed to be relatively small, i.e., counts of 10 or less in each cell. A statistically significant *p*-value, defined as *p*-value less than 0.05, generated by any of these tests would indicate a potentially substantial difference in the data distribution between any of the sub-cohorts. In addition, standardized mean differences (SMDs) were also calculated to assess the magnitude of differences between the sub-cohorts, where SMDs equal to 0.2, 0.5, and 0.8 represent small, medium, and large differences between cohorts, respectively [30]. 

#### Analysis of treatment change

Number of treatment changes and type of first treatment change within the follow-up period were reported (from Subset A), which were further stratified by comorbidities in a post-hoc analysis. Kaplan-Meier survival analysis (from the Main Cohort) was used to obtain a more robust and generalizable estimate of treatment duration in the real-world. Patients were followed up to the point of first treatment change (treatment discontinuation, treatment switch, treatment add-on, or treatment drop) or censoring (for patients who did not experience the event by end of the study follow-up period or who were lost to follow-up), whichever was earlier. Censoring-adjusted incidence rate of treatment change over the follow-up period was subsequently estimated.

Cox proportional-hazard models were developed on the Main Cohort to determine if any demographic and clinical characteristics were predictive of time to change in ADHD treatment. Relevant predictors were selected by first conducting a univariate analysis where individual characteristics were independently assessed for their statistical significance against the time to ADHD treatment change. Stepwise regression was then conducted where predictors were sequentially added into the model based on their statistical significance, measured using Wald’s test at a significance level of 0.05. To prevent predictors with strong multicollinearity against each other from being included in the model, the variance inflation factor (VIF) method was used after each step of the stepwise regression algorithm, where predictors with VIF exceeding 5 were excluded. Proportional hazard assumptions were examined, and interaction terms were explored. Unadjusted and adjusted hazard ratios, 95% confidence intervals (CI), and *p*-value (calculated using Wald’s test) of all predictors are reported to assess for the significance, relationship, and extent of contribution of each of the predictors to the change in treatment pattern. A variable with a hazard ratio (HR) of more than 1 and a *p*-value of < 0.05 represents that there is a higher risk of the experiencing treatment change related to the variable. Interactions between index ADHD medication against demographic variables and comorbidities were explored, and any interactions found to be statistically significant based on Wald’s test, i.e., *p*-value below 0.05, were included in the final regression model.

In addition, segmentation of the Main Cohort was done using classification tree analysis, which allowed us to understand not just the individual characteristics but also combination of characteristics that contribute to treatment change. The dependent variable was a binary outcome of whether treatment change was observed within 12 months from the index date.

#### Analysis of treatment response outcome

##### Assessment of HCRU rates

All analyses involving HCRU are conducted from Subset B. Frequency of inpatient hospitalization, outpatient visits, and composite HCRU rates (defined by a combination of inpatient, outpatient, and emergency department visits) were studied. Frequency of inpatient hospitalizations and outpatient visits were assessed up to 3, 6, 9, and 12 months after the index date and compared to frequency within 3 months before the index date using a Wilcoxon signed-rank test. Bonferroni correction was conducted to reduce the risk of Type I errors. Composite HCRU rates were compared for pre-index (3 and 6 months before index date) and post-index (3 and 6 months after index date) data using Wilcoxon signed-rank test. For both analyses, a significance level of 0.05 was used.

Generalized linear model with negative binomial distribution and log link function was used to assess the utilization frequency of psychiatric care resources during the follow-up period. Composite rates were used as the dependent variable to ensure sufficient variability in the data for regression modelling. Pre-index HCRU rates were included as an adjustment factor in the model. Variability in observation length was adjusted by introducing a time variable as an offset to model rates. All relevant predictors included in the final model for time to treatment change were subsequently considered as covariates to assess the association between patient characteristics and treatment response. Incidence rate ratios (IRRs) with 95% CI are reported for each covariate.

##### Assessment of disease severity

Disease severity was assessed at baseline and at 3-, 6-, 9-, and 12-months after the index date using subsets of patients from the Main Cohort with available CGI-S data at the relevant time points. Clinically meaningful and substantial improvements were defined as a decrease in CGI-S scores of at least 1 point and 2 points, respectively [31]. Wilcoxon signed-rank test was used to compare CGI-S scores at the index date and during follow-up. Bonferroni correction was conducted to reduce the risk of Type I errors. In addition, association between the baseline characteristics and change in CGI-S from index date to follow-up patients were studied on the Main Cohort with available CGI-S data using mixed linear models. As with the HCRU rates, covariates selected in the final treatment change model were also used as fixed effects in the mixed linear models, except for baseline CGI-S (encoded as an ordinal variable with 7 levels), which was applied as a random effect.

##### Software

Python 3.8 and R 4.0.3 were used for all analyses in this study.

## Results

### Patient demographic and clinical characteristics

**Fig. 2:**
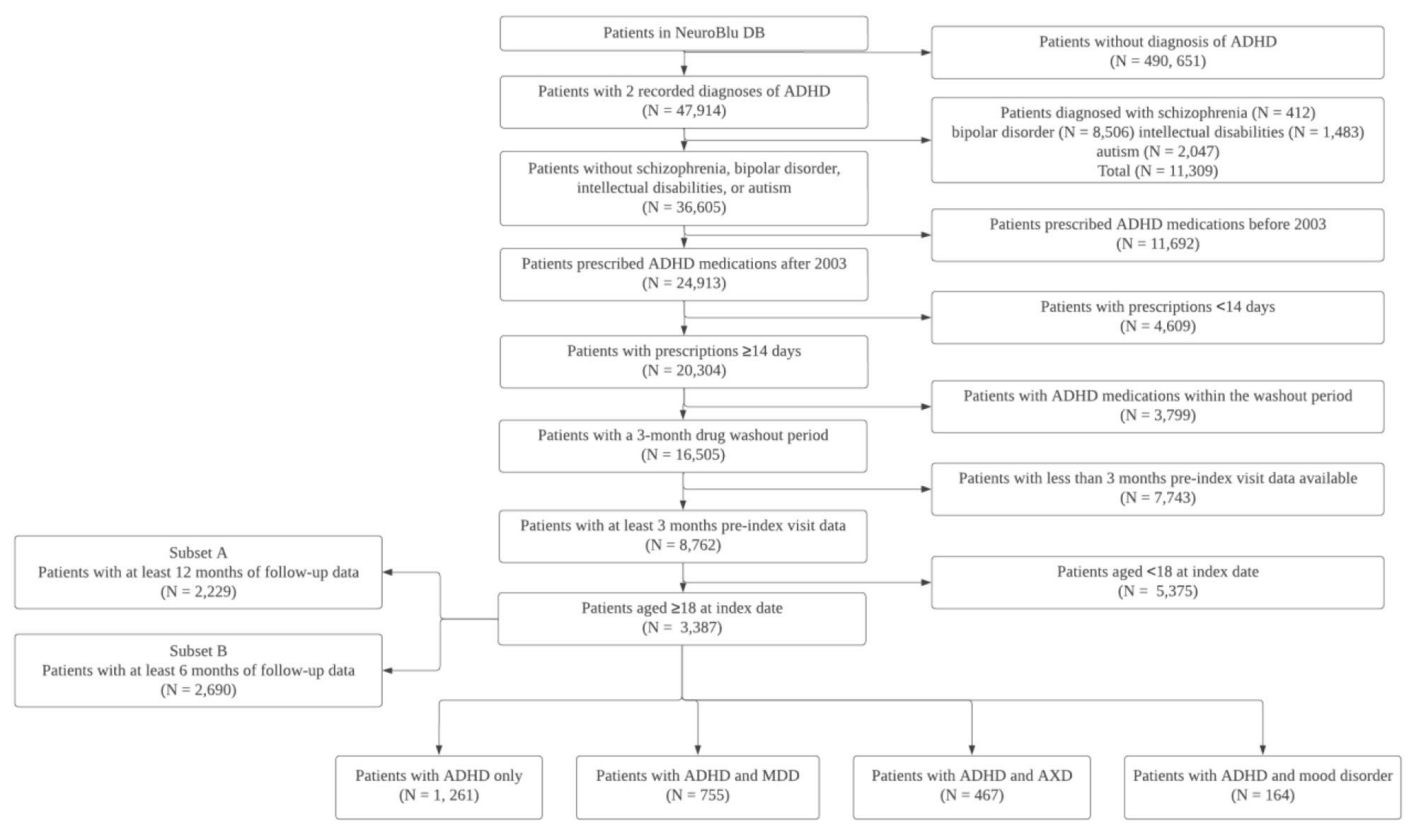
Attrition diagram for the Main Cohort and associated sub-cohorts **Abbreviations**: ADHD, attention-deficit/hyperactivity disorder; AXD, anxiety disorder; DB, database; MDD, major depressive disorder **Note**: Sub-cohorts do not add up to the Main Cohort (*N* = 3387). This is because there may be some patients who are multimorbid or patients with comorbidities unspecified (e.g., ADHD and PTSD)

**Fig. 3:**
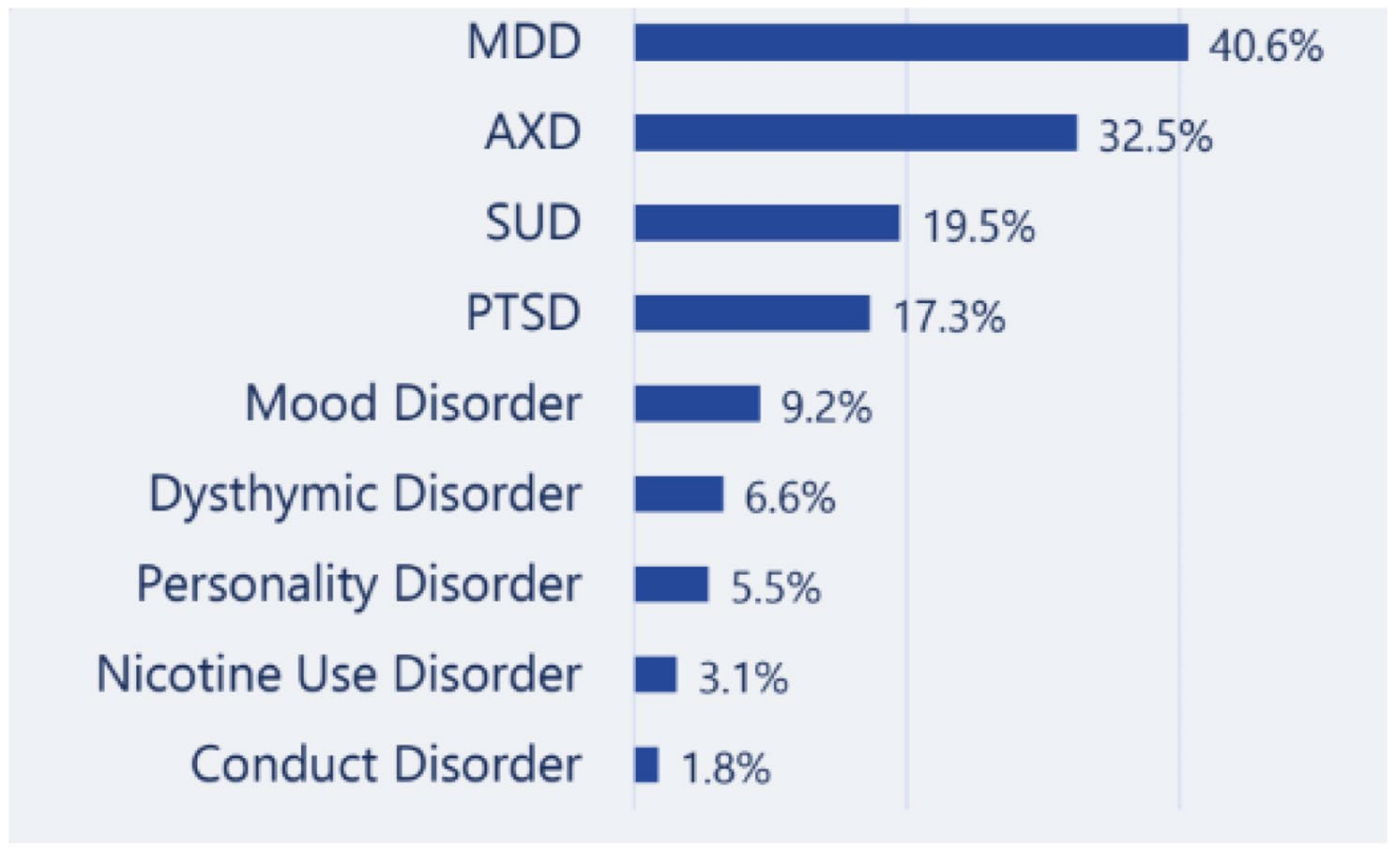
Top occurring psychiatric comorbidities at baseline among patients with ADHD **Abbreviations**: ADHD, attention-deficit/hyperactivity disorder; AXD, anxiety disorder; MDD, major depressive disorder; SUD, substance use disorder; PTSD, post-traumatic stress disorder

After applying study eligibility criteria, 3,387 patients were included in the Main Cohort (Fig. 2). This included 1,261 patients with ADHD only, 755 patients with ADHD and MDD, 467 patients with ADHD and anxiety disorder, and 164 patients with ADHD and mood disorder. Baseline demographic and clinical characteristics are reported in Table 1. The mean (SD) age of patients in the Main Cohort was 35.7 (12.6) years, 57.5% were female, and 67.5% were White. Most patients presented with the inattentive ADHD subtype (58.8%). Differences in baseline characteristics between cohorts were small (SMD < 0.5) for variables including age, sex, race, region, disease subtype, psychiatric comorbidities at baseline, and medications prescribed at baseline, except for antidepressants (SMD = 0.554). The top occurring psychiatric comorbidities at baseline were MDD (40.6%), anxiety disorder (32.5%), substance use disorder (SUD; 19.5%), and post-traumatic stress disorder (PTSD; 17.3%; Fig. 3). At index, most patients (89.6%) were prescribed stimulants. Among patients who were prescribed stimulants, approximately two thirds received long-acting stimulants (62.3%) and approximately one third received short-acting stimulants (31.3%). Other psychiatric medications prescribed concomitantly at index include antidepressants (55.3%) and anxiolytics (26.0%). At index, “issues with insight” was the top symptom, affecting 90.6% of patients, followed by “judgement”, affecting 19.2% of patients. All other symptoms were reported to occur in < 15% of patients. There were no statistically significant differences in psychiatric comorbidities or medications prescribed at baseline between sub-cohorts.

**Table 1:** Baseline demographic and clinical characteristics of the Main Cohort and associated sub-cohorts

Characteristic	Main cohort (*N* = 3,387)	ADHD only (*n* = 1261)	ADHD and MDD (*n* = 755)	ADHD and anxiety disorder (*n* = 467)	ADHD and mood disorder (*n* = 164)	Test statistic (*p*-value)	SMD
Age, years mean (SD)	35.7 (12.6)	34.1 (11.9)	39.3 (13.4)	34.6 (11.7)	31.3 (12.5)	H = 101.8 (< 0.001)^f^	0.328
Sex, *n* (%)							
Male	1,438 (42.5)	625 (49.6)	244 (32.3)	206 (44.1)	81 (49.4)	χ2 = 59.6 (< 0.001)^g^	0.195
Female	1,949 (57.5)	636 (50.4)	511 (67.7)	261 (55.9)	83 (50.6)		
Race. *n* (%)							
White	2,286 (67.5)	810 (64.2)	503 (66.6)	309 (66.2)	126 (76.8)	NA (< 0.001)^h^	0.298
Black/African American	115 (3.4)	38 (3.0)	31 (4.1)	9 (1.9)	12 (7.3)		
Native Alaskan/ American Indian/ Hawaiian/ Pacific Islander	20 (0.6)	9 (0.7)	3 (0.4)	2 (0.4)	0 (0.0)		
Asian	15 (0.4)	7 (0.6)	1 (0.1)	2 (0.4)	2 (1.2)		
Others^a^	56 (1.7)	22 (1.7)	8 (1.1)	12 (2.6)	3 (1.8)		
Unknown	895 (26.4)	375 (29.7)	209 (27.7)	133 (28.5)	21 (12.8)		
Region, *n* (%)							
Northeast	265 (7.8)	78 (6.2)	49 (6.5)	24 (5.1)	25 (15.2)	NA (< 0.001)^h^	0.350
Midwest	2,509 (74.1)	1,003 (79.5)	569 (75.4)	360 (77.1)	100 (61.0)		
South	361 (10.7)	121 (9.6)	66 (8.7)	56 (12.0)	9 (5.5)		
West	226 (6.7)	56 (4.4)	63 (8.3)	24 (5.1)	29 (17.7)		
Unknown	26 (0.8)	3 (0.2)	8 (1.1)	3 (0.6)	1 (0.6)		
Disease subtype, *n* (%)							
Predominantly inattentive	1,990 (58.8)	727 (57.7)	482 (63.8)	263 (56.3)	70 (42.7)	NA (0.001)^h^	0.231
Predominantly hyperactive	53 (1.6)	17 (1.3)	11 (1.5)	10 (2.1)	3 (1.8)		
Combined	1,075 (31.7)	416 (33.0)	209 (27.7)	152 (32.5)	71 (43.3)		
Undetermined	269 (7.9)	101 (8.0)	53 (7.0)	42 (9.0)	20 (12.2)		
Psychiatric comorbidity at baseline, *n* (%)							
MDD	1,377 (40.6)	0 (0.0)	755 (100.0)	0 (0.0)	0 (0.0)	NA (< 0.001)^h^	NA
Anxiety disorder	1,100 (32.5)	0 (0.0)	0 (0.0)	467 (100.0)	0 (0.0)	NA (< 0.001)^h^	NA
Mood disorder	313 (9.2)	0 (0.0)	0 (0.0)	0 (0.0)	164 (100.0)	NA (< 0.001)^h^	NA
Binge eating disorder	4 (0.1)	0 (0.0)	0 (0.0)	0 (0.0)	0 (0.0)	NA	0.058
Nicotine use disorder	106 (3.1)	0 (0.0)	0 (0.0)	0 (0.0)	0 (0.0)	NA	0
Post-traumatic stress disorder	586 (17.3)	190 (15.1)	142 (18.8)	66 (14.1)	26 (15.9)	χ2 = 6.5 (0.092)^g^	0.067
Substance use disorder	660 (19.5)	177 (14.0)	135 (17.9)	81 (17.3)	40 (24.4)	χ2 = 14.3 (0.003)^g^	0.135
Conduct disorder	61 (1.8)	27 (2.1)	3 (0.4)	0 (0.0)	17 (10.4)	NA (< 0.001)^h^	0.289
Impulse disorder	68 (2.0)	26 (2.1)	9 (1.2)	9 (1.9)	9 (5.5)	χ2 = 12.7 (0.005)^g^	0.125
Dysthymic disorder	222 (6.6)	62 (4.9)	46 (6.1)	38 (8.1)	8 (4.9)	χ2 = 6.8 (0.078)^g^	0.075
Schizoaffective disorder	52 (1.5)	27 (2.1)	2 (0.3)	12 (2.6)	0 (0.0)	< 0.001^h^	0.151
Personality disorder	186 (5.5)	28 (2.2)	50 (6.6)	19 (4.1)	17 (10.4)	χ2 = 37.8 (< 0.001)^g^	0.193
Medications prescribed at baseline, *n* (%)							
Anxiolytics	881 (26.0)	201 (15.9)	190 (25.2)	181 (38.8)	27 (16.5)	χ2 = 107.9 (< 0.001)^g^	0.3
Antidepressants	1,872 (55.3)	358 (28.4)	570 (75.5)	297 (63.6)	85 (51.8)	χ2 = 466.4 (< 0.001)^g^	0.554
Smoking cessation drug (e.g., bupropion)	516 (15.2)	89 (7.1)	198 (26.2)	50 (10.7)	18 (11.0)	χ2 = 154.4 (< 0.001)^g^	0.269
Stimulant	3035 (89.6)	1,176 (93.3)	681 (90.2)	424 (90.8)	137 (83.5)	χ2 = 19.9 (< 0.001)^g^	0.158
Long-acting	2,110 (62.3)^b^	NA	NA	NA	NA		
Short-acting	1,059 (31.3)^b^	NA	NA	NA	NA		
Unknown	252 (7.4)^b^	NA	NA	NA	NA		
Non-Stimulant	411 (12.1)	109 (8.6)	88 (11.7)	47 (10.1)	32 (19.5)	χ2 = 20.1 (< 0.001)^g^	0.167
Long-acting	323 (9.5)^c^	NA	NA	NA	NA		
Short-acting	35 (1.0)^c^	NA	NA	NA	NA		
Unknown	77 (2.3)^c^	NA	NA	NA	NA		
MSE category^d,e^							
Psychomotor	338 (11.0)	NA	NA	NA	NA	NA	NA
Mood	211 (6.8)	NA	NA	NA	NA	NA	NA
Speech	184 (6.0)	NA	NA	NA	NA	NA	NA
Affect	66 (2.1)	NA	NA	NA	NA	NA	NA
Cognition	291 (9.4)	NA	NA	NA	NA	NA	NA
Attention and concentration	268 (8.7)	NA	NA	NA	NA	NA	NA
Cognition	170 (5.5)	NA	NA	NA	NA	NA	NA
Insights	2,793 (90.6)	NA	NA	NA	NA	NA	NA
Judgement	592 (19.2)	NA	NA	NA	NA	NA	NA
Attitude	211 (6.8)	NA	NA	NA	NA	NA	NA

Abbreviations: ADHD, attention-deficit/hyperactivity disorder; MDD, major depressive disorder; MSE, mental status examination; NA, not applicable; SD, standard deviation; SMD, standardized mean difference

**Notes**:

a. ‘Others’ refers to patients of single ethnic groups that are not specified above, e.g. Latin Americans, or those who are of mixed race

b. Subset of stimulant medication

c. Subset of non-stimulant medication

d. MSE data available +/- 30 days from the index date for 3,083 patients

e. The MSE is an assessment of appearance, behavior, speech, mood, thoughts, cognition, and insight that provides information on the nature of a patient’s clinical presentation. A previously published NLP model was developed by psychiatrists and machine learning experts to produce clinically meaningful labels from unstructured MSE data, which were transformed into structured labels to facilitate quantitative analysis of behavioral manifestations specific to the patients’ outcomes [Mukherjee 2020]

f. Kruskal-Wallis *H* test was applied to calculate the *p*-value (rounded to 3 decimal places)

g. Chi-square test was applied to calculate the *p*-value (rounded to 3 decimal places)

h. Fisher’s exact test was applied to calculate the *p*-value (rounded to 3 decimal places)

### Treatment change outcomes

Among patients in Subset A, 44.8% of patients experienced a treatment change within the 12-month follow-up period. Among patients who experienced treatment change, 81.8% only changed treatment once. Of the patients who experienced treatment change of their index treatment, 65.0% discontinued, 11.8% had an add-on ADHD treatment, 11.8% switched to a different ADHD treatment, and 11.3% dropped medications from their index ADHD treatment. The rate of treatment change was consistent across four comorbidity sub-cohorts, with just over half of patients in each sub-cohort experiencing no treatment change, approximately one-third experiencing one treatment change, and the remaining patients experiencing two or more treatment changes (Table 2). When evaluating the first treatment change among sub-cohorts, treatment discontinuation was the most common type of first treatment change across all sub-groups, although it occurred at a higher rate among patients with ADHD only (70.8%) compared to patients with ADHD and a comorbidity (range: 55.3–65.6%; Table 2). Treatment switch and add-on were more common in patients with ADHD and comorbid MDD and anxiety disorder (switch: 18.9%; add-on: 20.5%) compared to other cohorts (range for switch: 8.5–13.6%; range for add-on: 8.9–12.1%). Treatment drop was however the least common in patients with ADHD and comorbid MDD and anxiety disorder (5.3%) and the most common in patients with ADHD and comorbid MDD (14.6%; Table 2). Differences observed in the type of first treatment change between sub-cohorts were statistically significant (χ2 = 31.2; *p* < 0.001; SMD = 0.309; Table 2).

**Table 2:** Treatment change patterns among Subset A patients and associated comorbidity sub-groups

Outcome	Subset A Cohort^a^ (*n* = 2,229)	Subcohort: ADHD only (*n* = 863)	Subcohort: ADHD and anxiety disorder (*n* = 316)	Subcohort: ADHD and MDD (*n* = 487)	Subcohort: ADHD and MDD and anxiety disorder^b^ (*n* = 306)	Test statistic (*p*-value)	SMD
Number of treatment changes within 1-year post-index, *n* (%)						χ2 = 5.9 (0.749)^c^	0.095
No change	1,231 (55.2)	479 (55.5)	176 (55.7)	275 (56.5)	174 (56.9)		
1 change	816 (36.6)	318 (36.8)	113 (35.8)	180 (37.0)	103 (33.7)		
2 changes	121 (5.4)	46 (5.3)	16 (5.1)	18 (3.7)	21 (6.9)		
≥3 changes	61 (2.7)	20 (2.3)	11 (3.5)	14 (2.9)	8 (2.6)		
Type of first treatment change within 1-year post-index, *n* (%)						χ2 = 31.2 (< 0.001)^c^	0.309
Patients with ≥1 treatment change	(*n* = 998)	(*n* = 384)	(*n* = 140)	(*n* = 212)	(*n* = 132)		
Discontinuation	649 (65.0)	272 (70.8)	88 (62.9)	139 (65.6)	73 (55.3)		
Add-on	118 (11.8)	34 (8.9)	17 (12.1)	24 (11.3)	27 (20.5)		
Switch	118 (11.8)	38 (9.9)	19 (13.6)	18 (8.5)	25 (18.9)		
Drop	113 (11.3)	40 (10.4)	16 (11.4)	31 (14.6)	7 (5.3)		

Abbreviations: ADHD, attention-deficit/hyperactivity disorder; MDD, major depressive disorder; SMD, standardized mean difference

**Notes**:

a. Treatment change descriptives were assessed for a subset of adult patients from the Main Cohort with 12 months of follow-up data available (*N* = 2,229; Subset A; the Full Main Cohort is *N* = 3,387). The four sub-cohorts are mutually exclusive, but do not add up to 2,229, as the sub-cohorts may also exclude those with other comorbidities not specified (e.g. ADHD + PTSD)

b. All sub-cohorts examining multimorbid patients was conducted post-study completion, during the manuscript discussion phase

c. Chi-square test was applied to calculate the *p*-value (rounded to 3 decimal places)

**Fig. 4:**
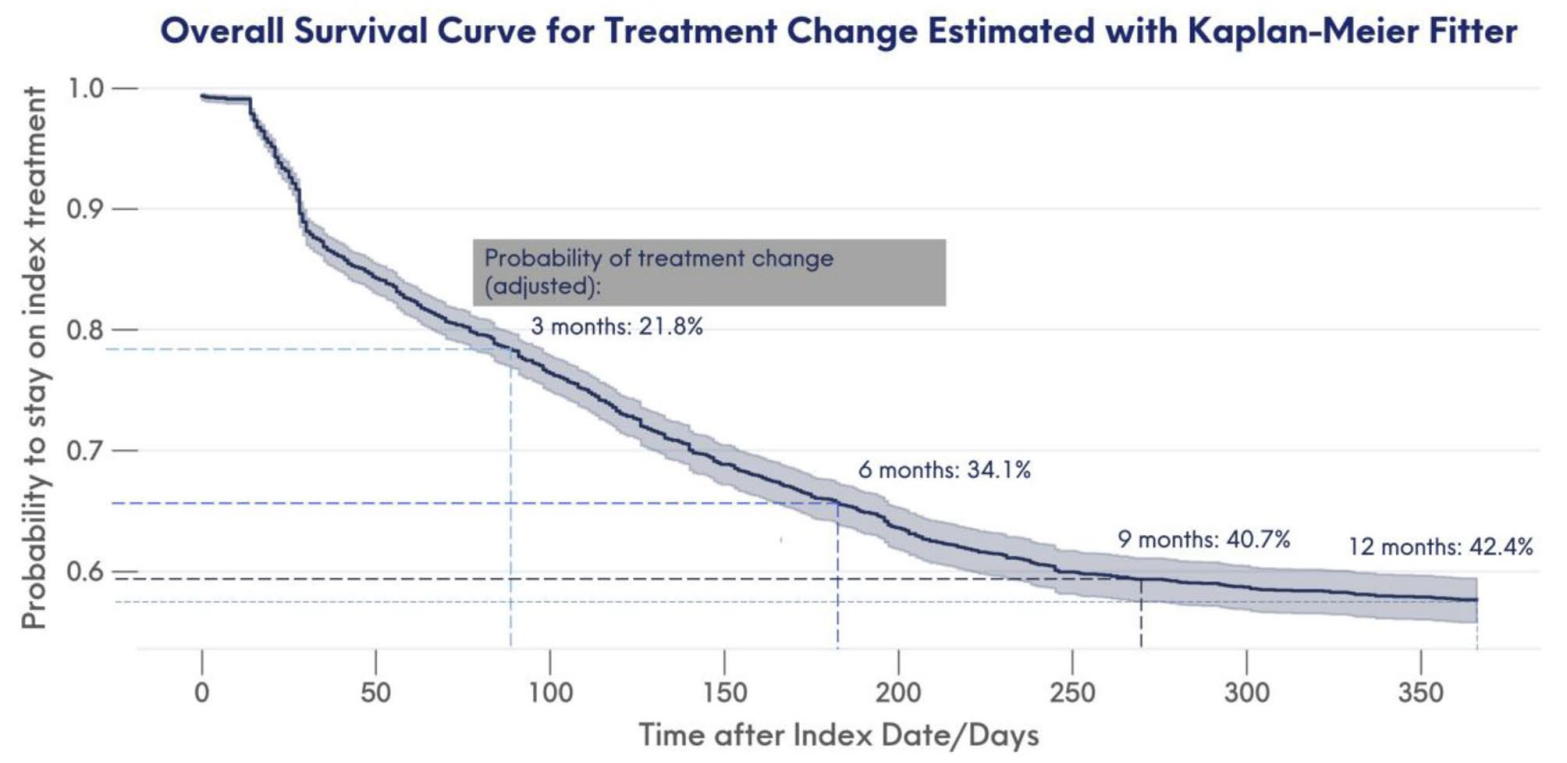
Survival curve of treatment change within the 12-month follow-up period

Survival analysis was also performed to provide a more robust estimate of treatment change using the Main Cohort, instead of Subset A. After accounting for censoring, the probability of treatment change was estimated to be 21.8% by the first three months of initiation, 34.1% by six months, and 42.4% by the end of the 1-year follow-up period (Fig. 4). Associations between baseline characteristics and time to treatment change results as derived from the regression analysis are reported in Table 3. After accounting all confounding variables selected using the stepwise selection, being prescribed both a stimulant and non-stimulant at index was significantly associated with increased risk of treatment change (adjusted HR: 1.64; 95% CI: 1.13, 2.38; *p* = 0.01; Table 3). Upon testing, there was no evidence of violations of the proportional hazards assumption.

**Table 3:** Time-to-treatment change - associations between baseline characteristics and time to treatment change in study

Characteristic		Main Cohort (*N* = 3,387)			
		Univariate Cox Regression		Multivariate Cox Regression	
		Crude HR (95% CI)	*p*-value^e^	Adjusted HR (95% CI)	*p*-value^e^
Age	10 years^b^	0.90 (0.86, 0.95)	< 0.001	0.90 (0.85, 0.94)	< 0.001*
Sex	Female	**Reference value**			
	Male	0.96 (0.86, 1.08)	0.53	0.92 (0.82, 1.04)	0.179
Race	White	**Reference value**			
	Black/African American	1.36 (1.04, 1.80)	0.027	1.29 (0.98, 1.70)	0.074
	Others^c^	0.98 (0.70, 1.38)	0.911	0.94 (0.67, 1.33)	0.737
	Unknown^d^	0.73 (0.63, 0.84)	< 0.001	0.75 (0.65, 0.86)	< 0.001*
Region	Northeast	0.91 (0.73, 1.14)	0.426	NA	NA
	Midwest	**Reference value**			
	South	1.34 (1.13, 1.59)	0.001	NA	NA
	West	1.45 (1.18, 1.77)	< 0.001	NA	NA
Baseline CGI-S	Mild	**Reference value**			
	Moderate	1.14 (0.98, 1.32)	0.096	NA	NA
	Severe	1.06 (0.77, 1.47)	0.728	NA	NA
Diagnosed psychiatric comorbidities at baseline	MDD	0.96 (0.85, 1.07)	0.452	0.97 (0.86, 1.09)	0.557
	Anxiety disorder	1.03 (0.92, 1.16)	0.583	1.03 (0.91, 1.16)	0.674
	Mood disorder	1.26 (1.06, 1.51)	0.011	1.12 (0.93, 1.35)	0.215
ADHD medication prescribed at index	Stimulant only	**Reference value**			
	Non-stimulant only	1.65 (1.40, 1.95)	< 0.001	0.89 (0.57, 1.40)	0.618
	Stimulant and non-stimulant	1.56 (1.07, 2.25)	0.019	1.64 (1.13, 2.38)	0.01*
Stimulant ingredients prescribed at index	Amphetamine	0.68 (0.61, 0.77)	< 0.001	NA	NA
	Methylphenidate	1.34 (1.17, 1.52)	< 0.001	NA	NA
	Modafinil	1.76 (0.97, 3.19)	0.061	NA	NA
Non-stimulant ingredients prescribed at index	Atomoxetine	1.73 (1.46, 2.05)	< 0.001	NA	NA
	Clonidine	1.54 (1.07, 2.21)	0.02	NA	NA
	Guanfacine	1.59 (1.02, 2.47)	0.04	NA	NA
Disease subtype	Combined	1.19 (1.05, 1.34)	0.005	NA	NA
	Predominantly hyperactive	1.34 (0.90, 2.00)	0.147	NA	NA
	Predominantly inattentive	**Reference value**			
	Others	0.97 (0.79, 1.21)	0.809	NA	NA
Interaction terms	Age x non-stimulant	NA	NA	1.02 (1.00, 1.03)	0.007*

Abbreviations: ADHD, attention-deficit/hyperactivity disorder; CGI-S, Clinical Global Impressions Scale – Severity; CI, confidence interval; HR, hazard ratio; MDD, major depressive disorder; MSE, mental status examination; NA, not applicable; SD, standard deviation

**Notes**:

a. Time to treatment change was assessed for the Main Cohort, which consists of all adult patients that meet the study eligibility criteria (*N* = 3,387)

b. Hazard ratio shown is for every 10-year increase in age

c. ‘Others’ refers to either patients of single ethnic groups that are not specified in the list, e.g., Latin Americans; OR those who are of mixed race

d. Race-related results should be interpreted with caution as there was a disproportionate number of patients with unknown race. To address this, sensitivity analysis (results not reported) was conducted on the multivariate Cox model with the race category removed, and results show that HRs for the remaining variables were largely unaffected

e. All *p*-values are generated using Wald’s test as part of the Cox proportional-hazard model development

Among the interaction effects studied, only interaction between age and index ADHD medication was found to be statistically significant (HR: 1.02; 95% CI: 1.00, 1.03; *p* = 0.007). For patients prescribed stimulants at index, every 10-year increase in age was associated with 10% decreased risk of treatment change (HR: 0.90; 95% CI: 0.85, 0.94). For patients prescribed non-stimulants at index, every 10-year increase in age was associated with 5% increased risk of treatment change (HR: 1.05; 95% CI: 0.95, 1.18), however the difference in risk was not statistically significant.

### Treatment response outcomes

#### Assessment of HCRU rates

Among patients in Subset B, the outpatient visit rate significantly increased from baseline (mean [SD] 1.03 [1.84] visits/month) to 3 months post-index (mean [SD] 1.62 [1.91] visits/month; *p* < 0.001), with visit rates declining steadily thereafter up to 12 months post-index (Supplemental Table 1). Factors that were found to be significantly associated with an increase in HCRU included being prescribed non-stimulant (IRR: 1.20; 95% CI: 1.04, 1.37; *p* = 0.01; ref: stimulant only) and having comorbid MDD (IRR: 1.16; 95% CI: 1.07, 1.26; *p* = 0.001) or mood disorder (IRR: 1.22; 95% CI: 1.07, 1.40; *p* = 0.004; Supplemental Table 2). Every 10 year increase in age was found to be associated with a 4% decrease in HCRU (IRR: 0.96; 95% CI: 0.93, 0.99; *p* = 0.009; Supplemental Table 2).

#### Assessment of disease severity

Within the first 3 months after index date, clinically meaningful improvements in CGI-S were observed in 26.6% of patients categorized with baseline CGI-S 4–5 (Supplemental Table 3) and 61% of patients in the Main Cohort categorized with baseline CGI-S 6–7 (Supplemental Table 4).

While controlling for all other variables, baseline CGI-S scores of 4–6 were associated with decrease in CGI-S (improvement; Supplemental Table 5) across the follow-up period. More frequent treatment change was associated with worsening CGI-S, although this finding was not statistically significant (Supplemental Table 6). Having comorbid MDD, anxiety disorder, or mood disorder was also found to be associated with slight worsening of CGI-S, although statistically insignificant.

## Discussion

In this retrospective real-world study, we examined the association of psychiatric comorbidities and patient characteristics with treatment change and treatment response in adult patients with ADHD. Among patients in the Main Cohort, psychiatric comorbidities including MDD, anxiety disorder, SUD, and PTSD were common, aligning with prior literature on adult ADHD which describes a heterogeneous population with several comorbidities [3, 25]. 

Our analysis did not find a statistically significant relationship between baseline comorbidities (MDD, anxiety disorder, or mood disorder) and ADHD treatment change, possibly because of the complexity of the comorbidities themselves not being accounted for in the model (e.g., duration of comorbid diagnosis, whether a patient was treated for comorbidities or not, and if so, the duration of treatment). Moreover, treatment change in this study was focused on ADHD treatment change rather than any treatment change (e.g., change of antidepressants or antipsychotics), thus the presence of comorbidities alone may not necessarily be sufficient to explain the occurrence of ADHD treatment change. Moreover, the interaction effect between the use of medication for a comorbidity and medication for ADHD was not studied in this analysis.

We did, however, find a significant interaction between patient age and index ADHD medication in predicting treatment change among adult patients with ADHD. These results indicate that the impact of ADHD medication on the likelihood of treatment change varies by age group, suggesting that younger and older adult patients may respond differently to specific medications or that treatment approaches may change as patients age and other conditions may arise. Indeed, patient age and medication type have been reported to influence medication adherence and persistence in patients with ADHD [32, 33]. Given that adult ADHD is understudied and underdiagnosed, this interaction warrants further study in future analyses.

Despite the lack of a significant association between comorbidities and treatment change, we found that nearly half of patients with ADHD experienced a treatment change within 1-year post index. This finding aligns with that of a recent large (*N* = 122,881) US claims-based study, which evaluated treatment patterns among adults with ADHD and found that approximately half (50.2%) of patients experienced a change in pharmacological treatment after an average treatment duration of 7.1 months [34]. The slight difference in proportions of patients experiencing treatment change between the previous study and the current study may be due to differences in the data sources (EHR vs. claims data) and study design. Furthermore, results from this study suggest that patients with ADHD and a psychiatric comorbidities such as MDD and anxiety disorder experience more treatment additions and switches compared to patients with ADHD alone, demonstrating the complexity of this patient population. These add to previous findings from a US-based claims analysis of 172,010 patients with ADHD, which found that adult patients with ADHD with comorbid anxiety disorder and/or depression experienced significantly higher odds of treatment change (odds ratio: 1.21; *p* < 0.05) compared to adults with ADHD without psychiatric comorbidities [27]. 

While we were not able to discern the reasons for treatment change using real-world data in this study, these findings suggest that current management and treatment patterns of ADHD may be inadequate at controlling symptoms. This hypothesis is supported by a separate US-based chart review study of 320 adult patients with ADHD, which found that reasons for treatment change included inadequate symptom control in over half (55.9%) of patients who discontinued, as well as factors such as occurrence of treatment-related complications, patient dislike of medication, and cost considerations [14]. Additionally, a systematic literature review of patients with ADHD found that the most commonly-reported reasons for ADHD medication discontinuation were ineffective symptom control and adverse effects [35]. In addition to the challenges that ADHD medication changes pose for the clinical management of ADHD, co-medication and polypharmacy are also a particular concern for more complex patient subgroups. In a recent study of adult patients with ADHD, the proportion of patients who received five or more medication classes ranged from 10% among patients aged 18 years to 60% among patients aged 64 years [36]. Additionally, the odds of being prescribed other psychotropic medications were substantially higher than for patients who did not receive ADHD medication [36]. 

ADHD also incurs a substantial economic and societal burden [34, 37], with one US-based study estimating total societal excess costs attributable to ADHD of $122.8 billion in 2018 [34]. In the current study, age was significantly associated with a slight decrease in HCRU, while being prescribed a non-stimulant and having a comorbid psychiatric condition (MDD or mood disorder) were both significantly associated with an increase in HCRU. The latter finding adds to results from previous studies in Sweden [38], Germany [39], and the US [40–42], which have reported that psychiatric comorbidities in patients with ADHD can lead to increased HCRU and costs in this patient population. We also observed a significant increase from baseline to 3 months post-index in outpatient visit rates among all patients, likely attributed to ADHD clinical management.

ADHD in adult populations is typically under-recognized and under-treated [18, 19]. Furthermore, few studies have evaluated the impact of psychiatric comorbidities on treatment patterns in this population, despite the high prevalence of comorbidities among patients with ADHD [22–24]. By using EHR-derived data, we were able to provide additional real-world evidence to support findings from previous claims analyses and chart-based studies that highlight the challenges patients with ADHD face, particularly with regards to treatment changes. As the prevalence of ADHD among adults increases [4], understanding the unmet treatment need is critical for development of effective novel therapeutic and management options.

Findings from this study are subject to limitations. First, there is the potential for unmeasured confounders and biases inherent to real-world data, as data collected from EHRs reflect real-world care patterns and vary in data quality and completeness. Second, as adherence and prescription fill data are not captured in the NeuroBlu Database, we assumed that patients followed through with their prescribed medication from documented start to end dates. This is a known limitation of using EHR-derived RWD and previous studies have used EHR data to examine treatment patterns and adherence [13, 43, 44]. Tracking and measuring adherence using administrative healthcare data (e.g., EHR data or claims data) has several known challenges and is a common limitation of real-world treatment pattern studies [45]. Third, as non-pharmacological data was not captured in the NeuroBlu Database at the time of this study, patients who received psychotherapy or other forms of behavioral therapy were not captured in this patient cohort. Psychotherapy is typically the first line of treatment for mental health disorders. Future studies could potentially explore the synergistic effect of pharmacotherapy and psychotherapy. Fourth, we were unable to capture patients who may have been prescribed ADHD and/or other relevant medications in the primary care setting, as their patient records are not reflected in the database. Fifth, approved pharmacological treatments for ADHD include short-acting and long-acting stimulants and non-stimulants. Indications for the type of formulation patients receive for their ADHD-related medication were only partially captured, therefore, treatment change modelling only considered treatment changes on the therapeutic agent level. ADHD-related medication categorized by type of formulation was only described using a simple summary for the present study. Sixth, modelling time-to-treatment change using Cox regression only accounts for patients’ first treatment change, afterwards the patient was censored from the study. Future studies may seek to further investigate the various treatment changes experienced across the study period using models that account for multiple events, such as multiple-event Cox models or competing risk models. Seventh, the use of composite utilization rates to gauge the overall healthcare resource utilization in this study does not entirely reflect the real-world economic burden experienced by patients due to the absence of cost data, limiting the interpretation of actual burden incurred. Eighth, the use of EHR data introduces the challenge of observing consistent patient resource utilization due to variations in patient encounters with the system, unlike those seen in clinical trials. Importantly, the issue of loss to follow-up is prevalent in EHR data, which reflects actual clinical practice in the real world. To address this, follow-up data requirements were designed to limit the impact of patient attrition, which could lead to an inherent selection bias, and bias caused by such variability, and therefore, our findings should be interpreted with these constraints in mind. Finally, findings from this study were associative, and causation cannot be inferred.

In conclusion, this real-world study provided evidence on an association of psychiatric comorbidities and patient characteristics on treatment change, treatment response, and HCRU in adult patients with ADHD. Together with existing literature, our findings support the need for future studies to examine the unmet medical and treatment needs of this complex patient population. Given the increasing incidence and prevalence of adult ADHD in the US, and ongoing shortages of front-line stimulant medication, continuous drug development could also identify and increase alternative treatment options to serve adult patients with ADHD with or without psychiatric comorbidities, potentially reducing the clinical and resource utilization burden observed in this study.

## Electronic supplementary material

Below is the link to the electronic supplementary material.

Supplementary Material 1

## Data Availability

The data that support the findings of this study are available from Holmusk Technologies, Inc., but restrictions apply to the availability of these data, which were used under license for the current study, and so are not publicly available. Data are however available from the authors upon reasonable request and with permission of Holmusk Technologies, Inc., and are subject to license agreement with Holmusk Technologies, Inc. Interested parties should contact publications@holmusk.com to determine licensing terms.

## Abbreviations

ADHD: Attention-deficit hyperactivity disorder
CBT: Cognitive behavioral therapy
CDM: Common Data Model
CGI-S: Clinical Global Impression – Severity
ED: Emergency department
EHR: Electronic health record
FDA: US Food & Drug Administration
HCRU: Healthcare resource utilization
HIPAA: Health Insurance Portability and Accountability Act
HR: Hazard ratio
ICD: International Classification of Diseases
IRR: Incidence rate ratio
IQR: Interquartile range
IRB: Institutional Review Board
MDD: Major depressive disorder
MSE: Mental State Examination
NLP: Natural language processing
OHDSI: Observational Health Data Sciences and Informatics
SD: Standard deviation
SUD: Substance use disorder
US: United States
VIF: Variance inflation factor
