# Supplementary material for: Real world analysis of treatment change and response in adults with attention-deficit/hyperactivity disorder (ADHD) alone and with concomitant psychiatric comorbidities: results from an electronic health record database study in the United States
Source: BMC Psychiatry. 2024 Sep 16;24:618. doi: 10.1186/s12888-024-05994-8 (PMC11406735; doi:10.1186/s12888-024-05994-8)
Supplement: Supplementary file 1 — Supplementary Material 1 [file 12888_2024_5994_MOESM1_ESM.docx]

**Real world analysis of treatment change and response in adults with attention-deficit/hyperactivity disorder (ADHD) alone and with concomitant psychiatric comorbidities: Results from an electronic health record database study is the United States**

# Additional Files

**Supplemental Table 1.** HCRU visit rate from baseline to follow-up^a^

| **Variable** | **Statistic** | **Baseline rate^b^** | **Follow-up rate** | **p-value** |
| --- | --- | --- | --- | --- |
| **HCRU - Inpatient rate (visits/month)** | | | | |
| Baseline^b^ vs. Months 0–3 | Rate | 0.021 (0.089) | 0.023 (0.109) | 0.651 |
| Baseline vs. Months 4–6 |  | 0.021 (0.089) | 0.01 (0.096) | 0.042 |
| Baseline vs. Months 7–9 |  | 0.021 (0.089) | 0.008 (0.057) | 0.011 |
| Baseline vs. Months 10–12 |  | 0.021 (0.089) | 0.012 (0.096) | <0.001 |
| **HCRU - Outpatient rate (visits/month)** | | | | |
| Baseline^a^ vs. Months 0–3 | Rate | 1.03 (1.84) | 1.62 (1.91) | <.001 |
| Baseline vs. Months 4–6 |  | 1.03 (1.84) | 1.13 (1.83) | <0.001 |
| Baseline vs. Months 7–9 |  | 1.03 (1.84) | 1.03 (1.73) | <0.001 |
| Baseline vs. Months 10–12 |  | 1.03 (1.84) | 0.97 (1.64) | 0.002 |
| **HCRU - Composite measure (visits/month)** | | | | |
| Months -3–0 vs. Months 0–3 | Rate | 1.19 (1.87) | 2.01 (1.9) | <0.001 |
| Months -6–0 vs. Months 0–6 |  | 1.18 (1.72) | 1.68 (1.73) | <0.001 |

**Abbreviations:** HCRU, healthcare resource utilization.

**Notes:**

1. HCRU change was assessed for a subset of adult patients from the main cohort with 12 months of follow-up data available (N = 2,229; Subset A).
2. Baseline refers to a period of Month -6 to Month 0.

**Supplemental Table 2.** Negative binomial model (HCRU)^a^

|  |  | **Subset B (N = 2,690)** | | | |
| --- | --- | --- | --- | --- | --- |
|  |  | **Univariate Model** | | **Multivariate Model** | |
| **Variable** | **Value** | **Unadjusted IRR (95% CI)** | **p-value** | **Adjusted IRR**  **(95% CI)** | **p-value** |
| **Pre-index** | Pre-index HCRU rate^b^ | 1.25 (1.23 - 1.27) | <.001 | 1.26 (1.24, 1.29) | <0.001* |
| **Age** | 10 years | 0.99 (0.99 - 0.99) | 0.002 | 0.96 (0.93, 0.99) | 0.009* |
| **Sex** | Female | **Reference value** | | | |
|  | Male | 1.04 (0.96 - 1.11) | 0.34 | 1.04 (0.96, 1.12) | 0.371 |
| **Race** | White | **Reference value** | | | |
|  | Black/African American | 1.10 (0.92, 1.32) | 0.294 | 0.94 (0.76, 1.18) | 0.61 |
|  | Others | 1.32 (1.03 - 1.72) | 0.031 | 1.03 (0.81, 1.32) | 0.794 |
|  | Unknown | 0.69 (0.63 - 0.75) | <0.001 | 0.84 (0.76, 0.92) | <0.001* |
| **Region** | Northeast | 2.06 (1.83 - 2.33) | <0.001 | - | - |
|  | Midwest | **Reference value** | | | |
|  | South | 1.20 (1.08 - 1.33) | <0.001) | - | - |
|  | West | 1.34 (1.18 - 1.53) | <0.001 | - | - |
| **Baseline CGI-S** | Mild | **Reference Value** | | | |
|  | Moderate | 1.27 (1.17 - 1.36) | <0.001 | - | - |
|  | Severe | 1.41 (1.20 - 1.67) | <0.001 | - | - |
| **Psychiatric comorbidities  at baseline** | Diagnosis of MDD | 1.13 (1.05 - 1.21) | 0.001 | 1.16 (1.07, 1.26) | 0.001* |
|  | Diagnosis of AXD | 1.11 (1.03 - 1.20) | 0.005 | 1.04 (0.96, 1.13) | 0.346 |
|  | Diagnosis of  Mood Disorder | 1.37 (1.23 - 1.54) | <0.001 | 1.22 (1.07, 1.40) | 0.004* |
|  | Diagnosis of  Binge Eating Disorder | 1.18 (0.91 - 1.57) | 0.226 | - | - |
|  | Diagnosis of  Nicotine Use Disorder | 1.61 (1.36 - 1.92) | <0.001 | - | - |
|  | Diagnosis of  Post-traumatic Stress Disorder | 1.36 (1.25 - 1.48) | <0.001 | - | - |
|  | Diagnosis of  Substance Use Disorder | 1.69 (1.56 - 1.84) | <0.001 | - | - |
|  | Diagnosis of  Conduct Disorder | 1.24 (0.96 - 1.61) | 0.107 | - | - |
|  | Diagnosis of Impulse Disorder | 1.33 (1.07 - 1.70) | 0.01 | - | - |
|  | Diagnosis of  Dysthymic Disorder | 1.28 (1.12 - 1.47) | <0.001 | - | - |
|  | Diagnosis of  Schizoaffective Disorder | 2.45 (1.99 - 3.05) | <0.001 | - | - |
|  | Diagnosis of  Personality Disorder | 1.59 (1.41 - 1.81) | <0.001 | - | - |
| **Other medications prescribed  at baseline** | Analgesics | 1.39 (1.24 - 1.56) | <0.001 | - | - |
|  | Anti-dementia drugs | 1.18 (0.59 - 2.8) | 0.664 | - | - |
|  | Anti-Parkinson drugs | 1.25 (0.93 - 1.72) | 0.16 | - | - |
|  | Anticonvulsants | 1.41 (1.30 - 1.54) | <0.001 | - | - |
|  | Antidepressants | 1.23 (1.14 - 1.33) | <0.001 | - | - |
|  | Anxiolytics | 1.01 (0.93 - 1.09) | 0.584 | - | - |
|  | Atypical antipsychotics | 1.49 (1.36 - 1.64) | <0.001 | - | - |
|  | Hypnotics and sedatives | 1.06 (0.94 - 1.20) | 0.325 | - | - |
|  | Lithium | 1.12 (0.82 - 1.57) | 0.477 | - | - |
|  | Substance abuse drugs | 1.48 (1.17 - 1.91) | 0.002 | - | - |
|  | Typical antipsychotics | 1.78 (1.29 - 1.62) | <0.001 | - | - |
| **ADHD medication prescribed  at baseline** | Stimulant only | **Reference value** | | | |
|  | Non-stimulant only | 1.64 (1.48 - 1.83) | <0.001 | 1.20 (1.04, 1.37) | 0.01* |
|  | Stimulant and non-stimulant | 1.11 (0.85 - 1.46) | 0.465 | 1.06 (0.77, 1.47) | 0.72 |
| **Stimulant ingredients prescribed  at baseline** | Amphetamine | 0.92 (0.85 - 0.99) | 0.047 | - | - |
|  | Methylphenidate | 0.93 (0.85 - 1.02) | 0.121 | - | - |
|  | Modafinil | 0.76 (0.49 - 1.22) | 0.226 | - | - |
| **Non-stimulant ingredients prescribed  at baseline** | Atomoxetine | 1.44 (1.28 - 1.62) | <0.001 | - | - |
|  | Clonidine | 2.05 (1.65 - 2.56) | <0.001 | - | - |
|  | Guanfacine | 1.19 (0.89 - 1.64) | 0.266 | - | - |
| **Disease subtype** | Combined | 1.07 (0.99 - 1.16) | 0.072 | - | - |
|  | Predominantly hyperactive | 1.05 (0.82 - 1.37) | 0.684 | - | - |
|  | Predominantly inattentive | **Reference value** | | | |
|  | Others | 1.14 (1.01 - 1.29) | 0.047 | - | - |

**Abbreviations:** CI, confidence interval; HCRU, healthcare resource utilization; MDD, major depressive disorder, AXD, anxiety disorder; IRR, incidence rate ratio.

**Notes:**

1. Association between baseline characteristics and HCRU was assessed for a subset of adult patients from the main cohort with 6 months of follow-up data available (N = 2,690; Subset B) to avoid the results being biased by patients who drop out of the study prematurely.
2. Pre-index HCRU rate refers to cumulative composite HCRU rate 3 months before index date: defined as the total number of outpatient and inpatient visits over the 3-month period, normalized to a monthly (30-day) rate and computed on a per-patient basis.

**Supplemental Table 3.** CGI-S improvement from baseline for patients with baseline CGI-S 4-5 (N = 1,436)

| **CGI-S** | **No. of patients** | **Clinically meaningful improvement (≥1 point decrease)** | **Clinically substantial improvement (≥2 point decrease)** |
| --- | --- | --- | --- |
| Month 0 vs. 3 | 856 | 228 (26.6%) | 63 (7.4%) |
| Month 0 vs. 6 | 503 | 129 (25.6%) | 34 (6.8%) |
| Month 0 vs. 9 | 331 | 80 (24.2%) | 28 (8.5%) |
| Month 0 vs. 12 | 225 | 52 (23.1%) | 13 (5.8%) |

**Abbreviations:** CGI-S, Clinical Global Impressions – Severity.

**Supplemental Table 4.** CGI-S improvement from baseline for patients with baseline CGI-S 6-7 (N = 113)

| **CGI-S** | **No. of patients** | **Clinically meaningful improvement (≥1 point decrease)** | **Clinically substantial improvement (≥2 point decrease)** |
| --- | --- | --- | --- |
| Month 0 vs. 3 | 59 | 36 (61.0%) | 17 (28.8%) |
| Month 0 vs. 6 | 28 | 16 (57.1%) | 11 (39.3%) |
| Month 0 vs. 9 | 23 | 17 (73.9%) | 11 (47.8%) |
| Month 0 vs. 12 | 16 | 10 (62.5%) | 6 (37.5%) |

**Abbreviations:** CGI-S, Clinical Global Impressions – Severity.

**Supplementary Table 5.** Random effects estimates for CGI-S change

| **Random effect variable** | **Value** | **Effect Estimate (95% CI)** | | |
| --- | --- | --- | --- | --- |
|  |  | **4-6 month (n=1,730)^b^** | **7-9 month (n=1,473)^b^** | **10-12 month (n=1,309)^b^** |
| **Baseline CGI-S^a^** | 1 | 1.08 (1.04, 1.11) | 1.34 (1.30, 1.38) | 1.32 (1.27, 1.36) |
|  | 2 | 0.49 (0.46, 0.53) | 0.47 (0.43, 0.51) | 0.56 (0.52, 0.60) |
|  | 3 | 0.15 (0.11, 0.18) | 0.03 (-0.01, 0.07) | 0.01 (-0.03, 0.05) |
|  | 4 | –0.13 (-0.17, –0.10) | –0.23 (–0.27, –0.19) | –0.23 (–0.27, –0.19) |
|  | 5 | –0.43 (-0.46, –0.39) | –0.58 (–0.62, –0.53) | –0.50 (–0.54, –0.46) |
|  | 6 | –0.88 (-0.92, –0.85) | –0.98 (–1.02, –0.93) | –1.20 (–1.25, –1.16) |
|  | 7 | –0.13 (-0.19, –0.06) | –0.21 (–0.28, –0.14) | 0.13 (0.05, 0.20) |

**Abbreviations:** CGI-S, Clinical Global Impressions – Severity; CI, confidence interval.

**Notes:**

- - - - 1. Positive effect estimates are associated with less improvement (or worsening) in clinical severity, whereas negative effect estimates are associated with more improvement (or less worsening) in clinical severity

**Supplemental Table 6.** Fixed effects estimates for CGI-S change

| **Fixed effect variable** | **Value** | **Effect Estimate (95% CI)** | | | | | |
| --- | --- | --- | --- | --- | --- | --- | --- |
|  |  | **4-6 month (N = 1,730)^b^** | **p-value** | **7-9 month (N = 1,473)^b^** | **p-value** | **10-12 month (N = 1,309)^b^** | **p-value** |
| **Treatment change** | No change | Reference value | | | | | |
|  | 1 change | 0.04 (–0.09, 0.16) | 0.571 | –0.02 (–0.14, 0.09) | 0.694 | 0.07 (–0.05, 0.19) | 0.245 |
|  | 2 changes | 0.18 (–0.17, 0.53) | 0.309 | 0.16 (–0.09, 0.40) | 0.219 | 0.03 (–0.23, 0.28) | 0.835 |
|  | 3 or more changes | –0.21 (–2.05, 1.62) | 0.819 | –0.19 (–0.62, 0.25) | 0.395 | 0.17 (–0.15, 0.49) | 0.296 |
| **Age** | 10 years^c^ | 0.02 (–0.02, 0.06) | 0.261 | 0.01 (–0.03, 0.05) | 0.629 | 0.03 (–0.01, 0.07) | 0.162 |
| **Sex** | Female | Reference value | | | | | |
|  | Male | –0.05 (–0.14, 0.04) | 0.287 | –0.08 (–0.18, 0.01) | 0.093 | –0.07 (–0.18, 0.04) | 0.216 |
| **Race** | White | Reference value | | | | | |
|  | Black/African American | –0.01 (–0.24, 0.21) | 0.902 | –0.10 (–0.36, 0.17) | 0.471 | –0.26 (–0.55, 0.03) | 0.076 |
|  | Others^d^ | 0.28 (–0.02, 0.58) | 0.065 | 0.19 (–0.15, 0.53) | 0.273 | 0.14 (–0.27, 0.55) | 0.507 |
|  | Unknown | –0.00 (–0.11, 0.10) | 0.952 | 0.02 (–0.09, 0.14) | 0.722 | –0.07 (–0.20, 0.06) | 0.297 |
| **Baseline comorbidity** | MDD | –0.00 (–0.09, 0.09) | 0.959 | 0.07 (–0.03, 0.17) | 0.182 | 0.08 (–0.03, 0.19) | 0.171 |
|  | Anxiety disorder | 0.04 (–0.06, 0.13) | 0.44 | 0.01 (–0.09, 0.11) | 0.872 | 0.03 (–0.08, 0.14) | 0.594 |
|  | Mood disorder | 0.04 (–0.11, 0.19) | 0.59 | 0.08 (–0.08, 0.24) | 0.33 | 0.04 (–0.13, 0.22) | 0.632 |
| **ADHD medication prescribed at baseline** | Stimulant | Reference value | | | | | |
|  | Non-stimulant | **0.17 (0.03, 0.30)** | **0.016*** | **0.15 (0.00**, **0.30)** | **0.048*** | 0.06 (–0.11, 0.23) | 0.477 |
|  | Stimulant + non-stimulant | –**0.41 (**–**0.78,** –**0.05)** | **0.027*** | –0.15 (-0.56, 0.25) | 0.457 | 0.04 (–0.41, 0.50) | 0.852 |

**Abbreviations:** CGI-S, Clinical Global Impressions – Severity; CI, confidence interval; MDD, major depressive disorder.

**Notes:**

1. Factors that explain variations in CGI-S change from baseline to follow-up were assessed for the CGI-S cohort (N = 2,152), which is a subset of patients from the Main Cohort (N = 3,387) with two additional requirements on CGI-S records: 1. patients must have one record of CGI-S within 14 days before index date (inclusive); 2. patients must have at least two recorded measurements of CGI-S within the follow-up period of 12 months.
2. Sample size includes only patients who have at least one CGI-S measurement available in the specified time period in the CGI-S cohort.
3. Effect estimate shown is for every 10-year increase in age.
4. ‘Others’ refers to either patients of single ethnic groups that are not specified in the list, e.g. Latin Americans; OR those who are of mixed race.

**Supplemental Figures.** Treatment change definition schematics

**Supplemental Figure 1.** Treatment discontinuation


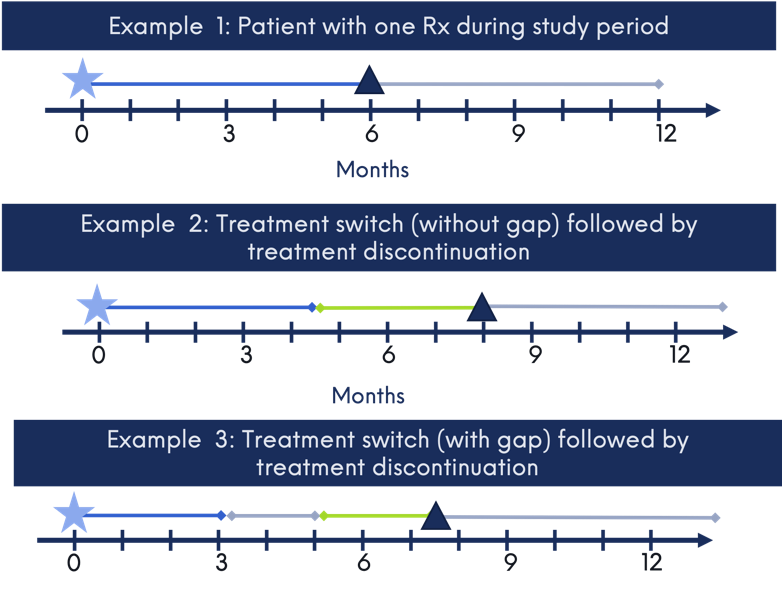


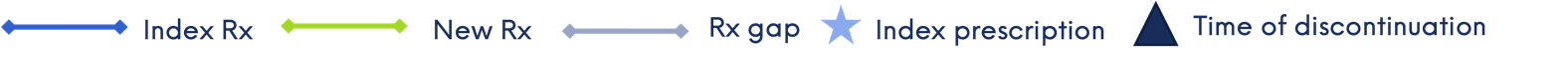


- In example 1, a discontinuation is confirmed by >4 months of no ADHD treatment.
- In example 2, the index treatment is swapped out for another treatment with no gaps. At Month 8, there is a cessation of treatment for >4 months. Patient is said to have discontinued at Month 8
- In example 3, patient has a short gap between index prescription and a new prescription (~2 months). Since the gap is shorter than 4 months, it is not considered to be a discontinuation. However, at Month ~7, patient stops all treatment for a longer period and is said to have discontinued.

**Figure 2.** Treatment switch


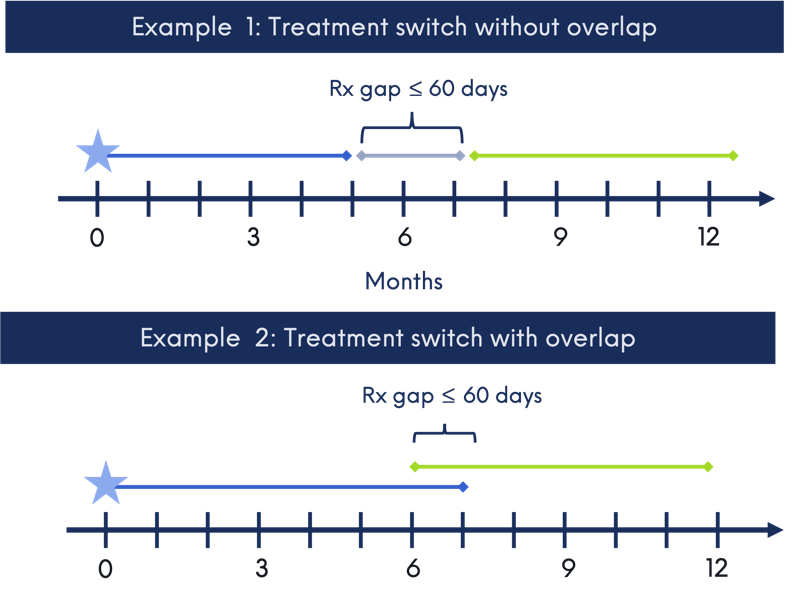


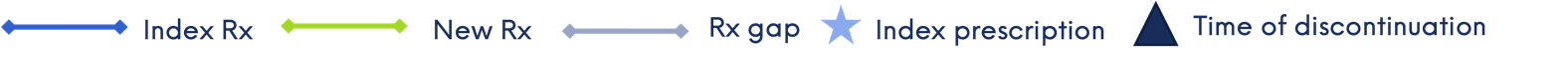


- In example 1, the patient’s index prescription ends, followed by a new prescription beginning shortly after (<60 days). This is determined to be a switch in treatment
- In example 2, the patient begins a new prescription before the index prescription ends. This is still considered to be a switch, as the overlap is <60 days.

**Figure 3.** Treatment add-on


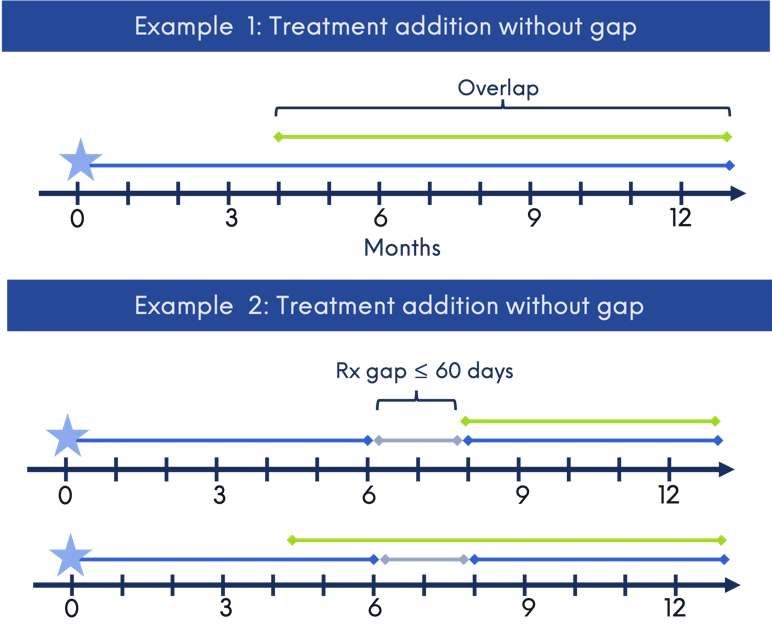


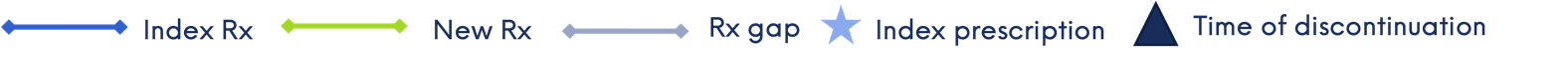


- In example 1, the patient has a second prescription in addition to their index prescription. Both persist for several months. This is considered an addition to index treatment.
- In example 2, patient adds on a new treatment after a short gap (<60 days). However, the index treatment persists after the short gap along with the new treatment. As such, the patient is considered to have a treatment add-on

**Figure 4.** Treatment drop


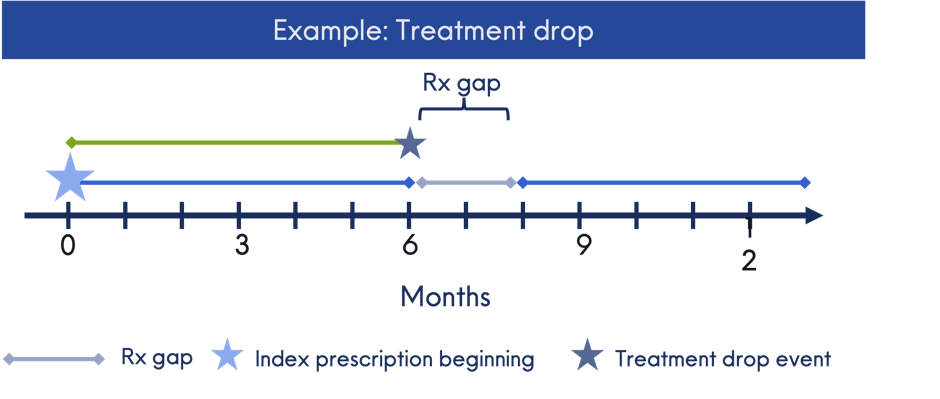


In the example above, it can be observed that patient dropped one of their index medications while continuing their other medication, this is considered a treatment drop.
